# The Effect of Aspirin on Preventing Vascular Access Dysfunction in Incident Hemodialysis Patients: A Prospective Cohort Study in Korean Clinical Research Centers for End-Stage Renal Disease (CRC for ESRD)

**DOI:** 10.3390/jcm8050677

**Published:** 2019-05-14

**Authors:** Chan Ho Kim, Hyung Jung Oh, Yon Su Kim, Yong-Lim Kim, Jae Hyun Chang, Dong-Ryeol Ryu

**Affiliations:** 1 Department of Internal Medicine, International St, Mary’s Hospital, Catholic Kwandong University College of Medicine, Incheon 22711, Korea; verdantk@daum.net; 2 Ewha Institute of Convergence Medicine, Ewha Womans University Mokdong Hospital, Seoul 07985, Korea; ohjmd@naver.com; 3 Department of Internal Medicine, Seoul National University of Medicine, Seoul 03080, Korea; yonsukim@snu.ac.kr; 4 Department of Internal Medicine, Kyungpook National University School of Medicine, Daegu 41944, Korea; ylkim@knu.ac.kr; 5 Department of Internal Medicine, Gachon University Gil Medical Center, Gachon University School of Medicine, Namdong Gu, Incheon 21565, Korea; 6 Department of Internal Medicine, School of Medicine, Ewha Womans University, Seoul 07985, Korea; 7 Tissue Injury Defense Research Center, Ewha Womans University, Seoul 07985, Korea

**Keywords:** aspirin, vascular access failure, incident hemodialysis

## Abstract

**Background:** Aspirin is often prescribed empirically to improve the patency of hemodialysis (HD) vascular access. Therefore, this study aimed to investigate the impact of aspirin on the survival of vascular access in incident HD patients with arteriovenous fistula (AVF) or arteriovenous graft (AVG). **Methods:** A prospective cohort of 881 incident HD patients was enrolled between 2009 and 2014. The primary outcome was defined as the first AVF/AVG intervention or salvage procedure, including percutaneous transluminal angioplasty or surgery for vascular access failure. Cox analyses were performed to determine the association between aspirin usage and the occurrence of the primary outcome. **Results:** The mean age of the patient group was 57.9 ± 13.4, and 63.8% of the patients were male. Aspirin was prescribed in 241 (27.4%) patients, and the median follow-up duration was 30 months. During follow-up, 180 (20.4%) patients experienced the primary outcome event. Univariate analysis showed that age, gender, presence of diabetes mellitus (DM), preexisting peripheral arterial disease, and the type of vascular access used (AVG versus AVF) were significantly associated with the development of the primary outcome. However, aspirin usage from the baseline was not significantly associated with primary outcome events (hazard ratio (HR): 1.16; 95% confidence interval (CI): 0.84–1.60; *p* = 0.378). Multivariate analysis showed that gender, the presence of DM, and the type of vascular access were still significantly associated with the occurrence of the primary outcome. Moreover, we did not observe the protective effect of taking aspirin on primary vascular access failure, even in subgroup analyses stratified according to gender, the presence of DM, and the type of vascular access. **Conclusion:** Physicians should carefully consider when they prescribe aspirin for the prevention of primary vascular access failure in Korean incident HD patients. In addition, larger prospective interventional studies are needed to elucidate the effect of aspirin on vascular access failure.

## 1. Introduction

Establishing and maintaining reliable vascular access is an important issue with respect to improving clinical care and outcomes in hemodialysis (HD) patients. Since arteriovenous fistula (AVF) and arteriovenous graft (AVG) are associated with a better clinical outcome, including infection and mortality, almost all physicians consider AVF and AVG as maintenance vascular access for HD [1,2,3,4]. However, vascular access failure is a common complication, and it is associated with substantial morbidity and mortality in patients requiring HD [5,6]. Thus, many nephrologists have focused on preventing and minimizing vascular access dysfunction. However, no definitive method exists to maintain long-term patency of vascular access.

Aspirin is an established antiplatelet drug used in the secondary prevention of cardiovascular disease caused by thrombotic events. It has been used safely in chronic kidney disease patients with careful monitoring of possible adverse effects such as gastrointestinal bleeding [7,8,9,10,11]. In addition, it has been proposed that aspirin has anti-oxidative and anti-inflammatory effects [12,13]. In contrast, recent studies suggest that neointimal hyperplasia and subsequent thrombosis, which are main causes of vascular access dysfunction, result from oxidative stress and inflammation [14,15]. Therefore, aspirin is often prescribed empirically to prevent vascular access failure in chronic HD patients. However, aspirin’s ability to inhibit neointimal hyperplasia and subsequent thrombosis in AVF or AVG is not conclusive [16,17,18].

The purpose of this study was to investigate the role of aspirin in preventing vascular access dysfunction in incident HD patients who began taking aspirin before HD initiation.

## 2. Materials and Methods

### 2.1. Subjects

All end-stage renal disease (ESRD) patients undergoing HD between May 2009 and December 2014 at one of the 36 centers of the Clinical Research Center for ESRD in Korea were recruited for this prospective, observational multicenter study. This study was part of a nationwide, multicenter, joint network, prospective cohort study on ESRD patients in Korea. It was designed to establish effective treatment guidelines, increase survival rates, and improve quality of life (clinicaltrial.gov NCT00931970).

**Figure 1:**
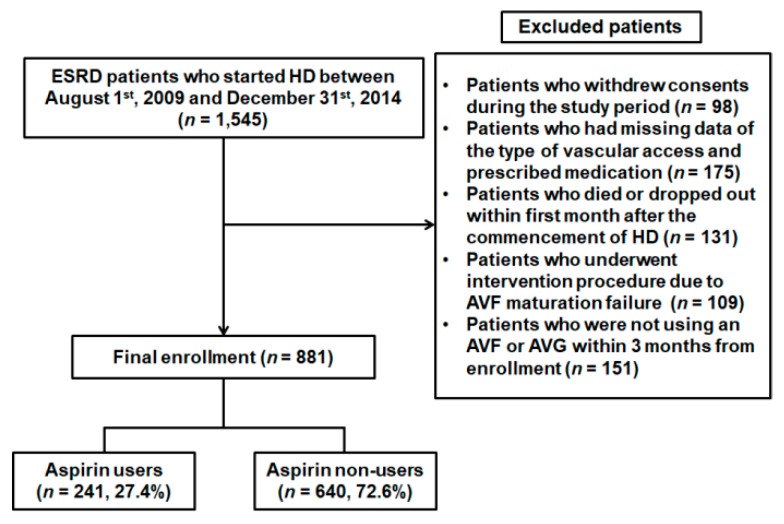
Flow diagram of the study subjects. From August 2009 to December 2014, 1545 patients who started HD at one of 36 centers of the CRC for ESRD in Korea were initially recruited for this prospective, observational multicenter study. According to the inclusion and exclusion criteria, 881 patients were included in the final analysis. Abbreviations; HD, hemodialysis; CRC for ESRD, clinical research center for end-stage renal disease; AVG, arteriovenous graft; DM, diabetes mellitus; AVF, arteriovenous fistula.

1545 patients who were at least 18 years of age and had started HD gave their consent between 1st August 2009 and 31st December 2014 to participate in the study. We excluded those who: Withdrew their consent during the study period (*n* = 98), had missing data regarding vascular access and prescribed medication (*n* = 175), died within the first month of initiation of HD (*n* = 131), underwent an intervention procedure due to AVF maturation failure (*n* = 109), and were not using an AVF or AVG within three months from the commencement of HD (*n* = 151). After these exclusions, a total of 881 patients were enrolled in the study, and the patients were monitored until 31st December 2015 (Figure 1).

The study protocol was approved by the Institutional Review Board of each participating center, and all patients provided written informed consent before their inclusion in the study.

### 2.2. Data Collection

Demographic information and clinical data such as age, gender, smoking status, and comorbidities were collected at the start of HD. Coronary arterial disease (CAD) was defined as a history of angina, myocardial infarction, coronary angioplasty, or coronary artery bypass grafts; cerebrovascular accident (CVA) was defined as a history of transient ischemic attack, stroke, or carotid endarterectomy; and, peripheral arterial disease (PAD) was defined as a history of claudication, any peripheral revascularization procedure, ischemic limb loss, and/or ulceration [19]. Each patient’s medication information was also investigated at baseline.

Additionally, we defined AVF maturation failure as an event requiring percutaneous or surgical intervention for the first use of AVF after vascular access creation. We also investigated why 151 patients could not use AVF or AVG within three months from HD initiation (Supplementary Table S1).

Laboratory data were gathered from blood samples of fasting patients before the start of HD in a midweek session. Hemoglobin, serum albumin, blood urea nitrogen (BUN), creatinine, total cholesterol, and triglycerides levels were measured. Body weight was recorded before dialysis on the same day that blood samples were drawn. Data were collected using a web-based platform of the Clinical Research Center for ESRD [20].

### 2.3. Outcome Measurement

Instances of vascular access failure and their date of occurrence were reported within one month of the event. The primary outcome was defined by the first AVF or AVG intervention or salvage procedure, including percutaneous transluminal angioplasty or surgery for vascular access failure. The secondary outcome was defined by the second AVF or AVG intervention or salvage procedure. Patients were censored at the time of kidney transplantation, failure to follow-up, or at the end of the follow-up period.

### 2.4. Statistical Analysis

Standard descriptive analyses were performed to compare the baseline demographics and clinical characteristics between aspirin users and non-users at the time of HD initiation. Continuous variables were expressed as mean ± standard deviation or median (interquartile ranges), and categorical variables were expressed as numbers with percentages. Patient characteristics were compared using the Student’s *t*-test or Mann–Whitney *U* test for continuous variables, and the Chi-square test was used for categorical variables. Cumulative incidence curves of the primary outcome were produced using the Kaplan-Meier method and compared with the log-rank test. The Cox proportional hazard model was also performed to assess whether aspirin usage was associated with the occurrence of the primary outcome. A multivariate Cox proportional analysis was conducted after adjusting for age, gender, the presence of diabetes mellitus (DM), preexisting PAD, and the type of vascular access used; these were the variables with a *p*-value < 0.05 in the univariate Cox analysis. A multivariate Cox proportional analysis was also conducted after adjusting for the presence of CAD and CVA, which were potential confounding covariates for vascular access dysfunction. For all survival analyses, the proportionality assumption of the Cox model was tested by visually examining the log-log survival plots. We also conducted subgroup analyses stratified by gender, the presence of DM, and the type of vascular access. To minimize the effects of age, gender, and the presence of DM, we performed propensity score (PS) matching with these variables. The PS for either the aspirin users or non-users was drawn by using multiple logistic regression analyses without considering the outcome. Using the Greedy 5- to 1-digit match algorithm, we constructed PS-matched pairs without replacement (1:1 match). More specifically, each subject in the aspirin user group was matched with an individual in the non-user group with a PS equal to 5 digits. If this was not the case, we used the method of sequentially pairing the next highest digit match (4-, 3-, 2-, or 1-digit match) in the PS to make the next best matches. After all PS matches were created, we evaluated the balance of baseline covariates between the 2 groups using paired *t* test for continuous variables and a McNemar test for categorical variables. To evaluate the association between aspirin usage and the secondary outcome, logistic regression analysis was performed. The results were presented as a hazard ratio (HR) for the Cox regression analysis, odds ratio (OR) for the logistic regression analysis, and 95% confidence intervals (CIs). Statistical analyses were conducted using the SPSS statistical package for Windows Ver. 20.0 (SPSS, Inc., Chicago, IL, USA) and SAS version 9.2 (SAS Inc., Cary, NC, USA). All tests were two-sided, and *p*-values < 0.05 were considered statistically significant.

## 3. Results

### 3.1. Baseline Characteristics

The demographic and clinical characteristics of the patients stratified according to aspirin usage are shown in Table 1. The mean age of the study group was 57.9 ± 13.4 years, and 63.8% (*n* = 562) of the patients were male. Aspirin was prescribed in 241 (27.4%) patients, and DM was presented in 540 (61.3%) patients. In addition, 126 (14.3%) patients had CAD, 88 (10.0%) patients were suffering from PAD, and 27 (3.1%) patients were diagnosed with CVA. There were 413 patients who were current or ex-smokers. The means of the hemoglobin, serum albumin, BUN, and creatinine measurements were 8.9 ± 1.6 g/dL, 3.4 ± 0.6 g/dL, 87.2 ± 37.7 mg/dL, and 8.9 ± 4.6 mg/dL, respectively. In addition, the primary outcome was developed in 180 (20.4%) patients during the median follow-up duration of 30 months.

The aspirin user group was significantly older (62.6 ± 11.3 vs. 56.2 ± 13.7 years, *p* <0.001) and had significantly more DM, CAD, PAD, and CVA when compared to the aspirin non-user group. Moreover, the aspirin user group contained significantly more current and ex-smokers. In contrast, there were more patients who were using AVF in the aspirin non-user group compared to the aspirin user group (81.0% vs. 75.9%, *p* = 0.018). The primary outcome occurred in 21.6% of the aspirin users, and it occurred in 20.0% of the aspirin non-users. This difference is not statistically significant. The incidence rate was also comparable between the two groups (0.091 vs. 0.073 person-year, *p* = 0.207). In addition, laboratory data showed no significant differences between the two groups except for total cholesterol. Total cholesterol levels were significantly lower in aspirin users than that in aspirin non-users (146.5 ± 43.1 vs. 158.4 ± 49.1 mg/dL, *p* = 0.002).

### 3.2. The Association between Aspirin Use and the Incidence of Vascular Access Failure

**Figure 2:**
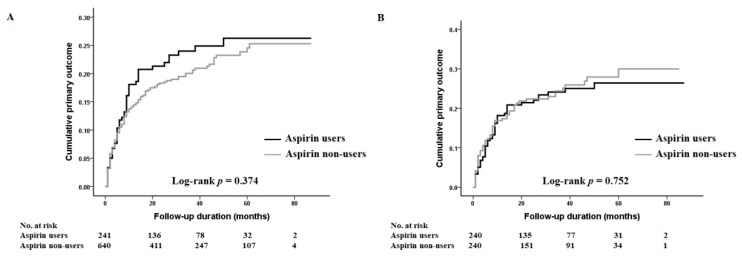
Kaplan-Meier plots for cumulative primary outcome; (**A**) overall subjects (**B**) propensity-matched cohort. There was no significant association between aspirin usage and protection against primary vascular access failure.

Table 2 shows the association between the variables and the incidence of vascular access dysfunction. In univariate analysis, age, gender, the presence or absence of DM, preexisting PAD (vs. non-PAD), and the type of vascular access used (AVG versus AVF) were significantly associated with the occurrence of primary vascular access failure (age, HR: 1.01, 95% CI: 1.00–1.02, *p* = 0.031; female, HR: 1.60, 95% CI: 1.20–2.15, *p* = 0.002; the presence of DM, HR:1.88, 95% CI: 1.35–2.62, *p* <0.001; preexisting PAD, HR: 1.84, 95% CI: 1.24–2.74, *p* = 0.003; using AVG, HR: 1.77, 95% CI: 1.28–2.46, *p* = 0.001). However, we observed no protective effect regarding primary vascular access failure by taking aspirin (HR: 1.16; 95% CI: 0.84–1.60; *p* = 0.378) (Figure 2). In the multivariate analysis, gender, the presence or absence of DM, and the type of vascular access used were still significantly associated with the development of primary vascular access failure (female, HR: 1.68, 95% CI: 1.25–2.25, *p* = 0.001; the presence of DM, HR: 1.71, 95% CI: 1.21–2.41, *p* = 0.002; using AVG, HR: 1.55, 95% CI 1.11–2.16, *p* = 0.01) (Table 2). However, the HR for the incidence of primary vascular access dysfunction in aspirin users was 0.89 (95% CI; 0.62–1.27, *p* = 0.51), which suggests there was no significant association between aspirin usage and protection against primary vascular access dysfunction.

We also investigated the effect of aspirin use on the incidence of primary vascular access dysfunction of each group when sorted by gender, the presence of DM, and the type of vascular access. However, we could not find any benefit to taking aspirin for protection against primary vascular access failure even in these subgroup analyses (Table 3).

We further analyzed the association of aspirin use with the primary outcome using PS-matched data. Matching was conducted with covariates such as age, gender, and the presence of DM. Each group contained 240 patients (Supplementary Table S2). In the PS-matched cohort, preexisting PAD verses non-PAD and the type of vascular access used (AVG versus AVF) were significantly associated with occurrence of primary vascular access failure (preexisting PAD, HR: 1.79, 95% CI: 1.06–3.05, *p* = 0.031; using AVG, HR: 1.58, 95% CI: 1.03–2.42, *p* = 0.037). However, no significant association was observed between aspirin use and protection against primary vascular access failure (Table 4).

### 3.3. The Effect of Aspirin Usage on Secondary Vascular Access Failure

Among the 180 patients who had primary vascular dysfunction, 64 (35.6%) patients underwent a second procedure due to repeated vascular access failure (secondary outcome). In the analysis that was restricted to patients with the primary outcome, the use of aspirin did not reduce the risk of incidence of the secondary outcome, either (OR: 1.34; 95% CI: 0.69–2.60; *p* = 0.389).

## 4. Discussion

AVF and AVG have been widely used for more than 50 years to create vascular access for chronic HD patients. Several studies have shown that all-cause mortality was significantly decreased in HD patients using AVF or AVG compared to those using a central venous catheter [1,2,4]. Therefore, AVF and AVG are now the preferred means of vascular access in chronic HD patients.

However, most instances of vascular access dysfunction result from venous stenosis or vascular thrombosis, which is more prevalent in HD patients with AVG compared to those with AVF [6]. Complications related to HD vascular access are serious in HD patients and include increased morbidity, mortality, and economic burden [6,21]. A safe and cost-effective management to prevent vascular access stenosis and subsequent thrombosis could lead to improved clinical outcomes for these patients. However, there are currently no definitive solutions for this clinical problem.

Several factors such as age, the presence of DM, higher body mass index, smoking status, history of cytomegalovirus infection, higher total cholesterol level, lower protein intake, the presence of PAD, and taking antiplatelet agents are considered risk factors for vascular access dysfunction [22,23,24,25,26]. Despite the heterogeneity of the factors related with vascular access survival, recent studies have revealed several major common pathways such as inflammation, oxidative stress, uremia, hypoxia, increased thrombogenicity, and shear stress that are responsible for progressive neointimal hyperplasia and propagating thrombosis [14,15]. Although the precise mechanisms and their interplay are not completely understood at present, some nephrologists have tried to elucidate aspirin’s protective effects on vascular access dysfunction.

Aspirin is the most commonly used antiplatelet agent and is suggested as medical therapy, which potentially inhibits neointimal hyperplasia and improves vascular access patency by influencing anti-inflammation, anti-oxidative stress, and platelet aggregation inhibition mechanisms [12,13,27,28,29]. However, the effect of aspirin has not been adequately evaluated with respect to vascular access failure in incident HD patients. Thus, we used a large ESRD cohort database to examine the protective effects of aspirin use on primary vascular access failure. In many countries, including Korea, more than 80% of incident HD patients still initiate dialysis with a central venous catheter [3]. Therefore, we enrolled incident HD patients who were using AVF or AVG within three months from HD initiation.

We found that aspirin use itself, did not significantly improve the survival rates associated with vascular access (AVF or AVG) in both primary vascular access failure and secondary vascular access dysfunction. Patients treated with aspirin were older and had a higher burden of comorbidities, such as DM, CAD, PAD, and CVA. These differences might partially explain the lack of expected benefit from the use of aspirin. Gender, the presence of DM, and the type of vascular access used still exhibited significant associations with the survival of vascular access even after adjustment for several confounding variables, which is consistent with the results of other previous studies [29,30,31,32]. Therefore, we performed subgroup analyses stratified according to gender, the presence of DM, and the type of vascular access used to minimize their effects on aspirin’s potential impact on vascular access survival. A PS-matched analysis was also performed. However, neither the subgroup analyses nor the PS-matched analysis changed the insignificant relationship between baseline aspirin use and HD vascular access survival.

There have been several observational and interventional studies that suggest aspirin has a positive effect on vascular access survival for HD [27,28,29,31,33]. Hasegawa et al. reported consistent aspirin use was associated with improved AVF survival among incident HD patients [27]. However, this study was performed with patients enrolled between 1996 and 2004, and investigated continuous aspirin use. Dixon et al. showed that use of aspirin was related with a trend toward longer primary patency of newly placed AVG [28]. However, this study was restricted to patients with AVG. Locham et al. also presented the beneficial effect of aspirin in patients with AVG [29]. However, the follow-up duration of the study was relatively shorter than that of our study. In contrast, multiple studies, including recent meta-analysis studies, failed to show the benefit of aspirin in vascular access survival [16,17,34,35]. All taken together, this discrepancy may be due to the heterogeneity of the study design and clinical situation (e.g., the dose, length of follow-up, combination drug, and research period). Moreover, some studies even suggested that aspirin had a detrimental effect on vascular access patency [36,37]. Due to the fact that patients who receive antiplatelet agents, including aspirin, usually exhibit more severe comorbidities than patients who do not receive these drugs, previous studies may have been influenced by selection bias. Therefore, great care should be exercised when interpreting previous research results.

The role of adjuvant medications, apart from aspirin, in improving vascular access patency is currently an important research topic. Despite various studies involving medications like clopidogrel, dipyridamole, fish oil, warfarin, and statin, the effects of current medical treatments on vascular access survival are inconclusive [16,17,32,35,36,38]. Therefore, a well-designed randomized controlled trial is warranted to establish the role of these agents in HD vascular access survival.

The current study has several limitations. First, indications, doses, and the withdrawal of aspirin were not investigated. Since baseline information of aspirin prescription could not warrant its consistent use, bias may have been introduced into our study. The dose and indication of aspirin might also be confounding factors in the results. Second, the data of several unmeasured confounders like vascular diameter, the presence of vascular calcification, the location of the vascular access (forearm or upper arm), and DM control status are not available in the database of CRC for ESRD. It is possible that these factors might cause some residual bias. Third, medications such as clopidogrel, dipyridamole, fish oil, warfarin, and statin could affect the patency of vascular access. Therefore, more studies evaluating the combined effects of these drugs on vascular access survival are required. Despite these limitations, the fact that the results were derived from a large prospective cohort of uniform patient population with relatively long-term follow-up duration should be considered a positive point.

In conclusion, it is difficult to conclude whether aspirin treatment at the time of HD commencement reduces the incidence of primary and secondary vascular access failure in incident HD patients. Physicians should carefully consider using aspirin to prevent primary vascular access failure in incident HD patients. However, since the long-term efficacy of aspirin and other antiplatelet agent treatments in preventing HD vascular access failure due to neointimal hyperplasia and thrombosis has not yet been determined, further prospective randomized trials are needed to elucidate the role of antiplatelet agents, including aspirin, on HD vascular access survival.

## Figures and Tables

**Table 1:** Baseline clinical characteristics and biochemical variables of the study population according to baseline aspirin use.

Variables	Total (*n* = 881)	Aspirin Users (*n* = 241)	Aspirin Non-Users (*n* = 640)	*p*-Value
Age, years	57.9 ± 13.4	62.6 ± 11.3	56.2 ± 13.7	<0.001
Male, *n* (%)	562 (63.8%)	161 (66.8%)	401 (62.7%)	0.253
Body mass index, kg/m^2^	23.2 ± 3.5	23.2 ± 3.3	23.2 ± 3.5	0.815
Comorbid diseases, *n* (%)				
Diabetes mellitus	540 (61.3%)	176 (73.0%)	364 (56.9%)	<0.001
Coronary arterial disease	126 (14.3%)	81 (33.6%)	45 (7.0%)	<0.001
Peripheral arterial disease	88 (10.0%)	40 (16.6%)	48 (7.5%)	<0.001
Cerebrovascular accident	27 (3.1%)	12 (5.0%)	15 (2.3%)	0.043
Smoking, *n* (%)				
Non-smoker	468 (53.1%)	113 (46.9%)	355 (55.5%)	0.023
Ex- or Current smoker	413 (46.9%)	128 (53.1%)	285 (44.5%)	
Biochemical parameters				
Hemoglobin, g/dL	8.9 ± 1.6	9.1 ± 1.6	8.8 ± 1.6	0.073
Albumin, g/dL	3.4 ± 0.6	3.4 ± 0.6	3.4 ± 0.6	0.781
Blood urea nitrogen, mg/dL	87.2 ± 37.7	86.4 ± 35.0	88.5 ± 38.6	0.122
Creatinine, mg/dL	8.9 ± 4.6	8.7 ± 3.3	9.0 ± 4.9	0.085
Total cholesterol, mg/dL	155.2 ± 47.8	146.5 ± 43.1	158.4 ± 49.1	0.002
Triglyceride, mg/dL	124.7 ± 76.7	121.1 ± 71.3	126.1 ± 78.7	0.460
Arteriovenous fistula, *n* (%)	714 (81.0%)	183 (75.9%)	531 (83.0%)	0.018
Vascular access failure event (primary outcome) ‡, n (%)	180 (20.4%)	52 (21.6%)	128 (20.0%)	0.605
Follow-up duration, months †	30 (11–50)	26 (8–47)	31 (12–51)	0.033
Incidence rate, person-year	0.077	0.091	0.073	0.207

Data are expressed as mean ± SDs or numbers (%) except where noted. † Median (interquartile range). ‡ The primary outcome was defined by the first AVF/AVG intervention or salvage procedure, including percutaneous transluminal angioplasty or surgery for vascular access failure. Abbreviation; SD, standard deviation; AVF, arteriovenous fistula; AVG, arteriovenous graft.

**Table 2:** Cox proportional regression analysis for vascular access failure event (primary outcome).

Variables	Univariate		Multivariate	
	HR (95% CI)	*p*-Value	HR (95% CI)	*p*-Value
Age, per 1 year	1.01 (1.00–1.02)	0.031	1.01 (1.00–1.02)	0.332
Female vs. male	1.60 (1.20–2.15)	0.002	1.68 (1.25–2.25)	0.001
Body mass index, per 1 kg/m^2^	0.99 (0.95–1.04)	0.779	-	-
DM vs. non-DM	1.88 (1.35–2.62)	<0.001	1.71 (1.21–2.41)	0.002
Pre-existing CAD vs. non-CAD	1.41 (0.97–2.06)	0.072	1.33 (0.88–2.01)	0.179
Pre-existing PAD vs. non-PAD	1.84 (1.24–2.74)	0.003	1.45 (0.95–2.21)	0.086
Pre-existing CVA vs. non-CVA	1.79 (0.91–3.49)	0.090	1.73 (0.87–3.43)	0.118
Smoking				
Non-smoker	1.00 (reference)	-	-	-
Ex- or Current smoker	0.78 (0.58–1.05)	0.101	-	-
Total cholesterol, per 1 mg/dL	1.00 (0.99–1.01)	0.114	-	-
Triglyceride, per 1 mg/dL	1.00 (0.99–1.00)	0.106	-	-
AVG vs. AVF	1.77 (1.28–2.46)	0.001	1.55 (1.11–2.16)	0.010
Aspirin users vs. Aspirin non-users	1.16 (0.84–1.60)	0.378	0.89 (0.62–1.27)	0.510

Abbreviation; HR = hazard ratio; CI = confidence interval; DM = diabetes mellitus; CAD = coronary arterial disease; PAD = peripheral arterial disease; CVA = cerebrovascular accident; AVG = arteriovenous graft; AVF = arteriovenous fistula.

**Table 3:** Multivariate Cox proportional regression analysis for vascular access failure event (primary outcome) in each stratified group †.

Subgroup	Aspirin Users vs. Aspirin Non-Users	
	HR (95% CI)	*p*-Value
Gender ‡		
Male	0.89 (0.55–1.45)	0.643
Female	0.86 (0.51–1.46)	0.571
Diabetes mellitus §		
Yes	0.94 (0.63–1.40)	0.767
No	0.92 (0.51–1.67)	0.408
The type of vascular access ¶		
AVF	0.83 (0.54–1.28)	0.392
AVG	1.11 (0.59–2.08)	0.750

† Patients were stratified according to gender, the presence of DM, and the type of vascular access. ‡ Adjusted for age, DM, CAD, PAD, CVA, and the type of vascular access. § Adjusted for age, gender, CAD, PAD, CVA, and the type of vascular access. ¶ Adjusted for age, gender, DM, CAD, PAD, and CVA. Abbreviation; HR = hazard ratio; CI = confidence interval; DM = diabetes mellitus; CAD = coronary arterial disease; PAD = peripheral arterial disease; CVA = cerebrovascular accident; AVF = arteriovenous fistula; AVG = arteriovenous graft.

**Table 4:** Cox proportional regression analysis for vascular access failure event (primary outcome) in the propensity-matched cohort.

Variables	Univariate		Multivariate	
	HR (95% CI)	*p*-Value	HR (95% CI)	*p*-Value
Age, per 1 year	1.00 (0.98–1.02)	0.914	-	-
Female vs. male	1.69 (1.16–2.45)	0.007	1.27 (0.78–2.09)	0.340
Body mass index, per 1 kg/m^2^	0.99 (0.94–1.05)	0.775	-	-
DM vs. non-DM	1.61 (1.01–2.56)	0.047	1.46 (0.91–2.36)	0.117
Pre-existing CAD vs. non-CAD	1.15 (0.74–1.78)	0.542	1.14 (0.70–1.85)	0.595
Pre-existing PAD vs. non-PAD	1.84 (1.15–2.97)	0.012	1.79 (1.06–3.05)	0.031
Pre-existing CVA vs. non-CVA	1.93 (0.94–3.96)	0.074	1.75 (0.84–3.66)	0.139
Smoking				
Non-smoker	1.00 (reference)	-	1.00 (reference)	-
Ex- or Current smoker	0.76 (0.40–1.12)	0.135	-	-
Total cholesterol, per 1 mg/dL	1.00 (0.99–1.01)	0.250	-	-
Triglyceride, per 1 mg/dL	1.00 (0.98–1.02)	0.193	-	-
AVG vs. AVF	1.59 (1.05–2.41)	0.029	1.58 (1.03–2.42)	0.037
Aspirin users vs. Aspirin non-users	0.94 (0.65–1.37)	0.754	0.84 (0.56–1.24)	0.376

Abbreviaiton; HR = hazard ratio; CI = confidence interval; DM = diabetes mellitus; CAD = coronary arterial disease; PAD = peripheral arterial disease; CVA = cerebrovascular accident; AVG = arteriovenous graft; AVF = arteriovenous fistula.

## Supplementary Materials

The following are available online at https://www.mdpi.com/2077-0383/8/5/677/s1, Table S1: Patients who were not using AVF or AVG within three months from the HD initiation, Table S2: Baseline clinical characteristics and biochemical variables of the study population according to baseline aspirin use after propensity score matching.
