# Supplementary material for: The Effect of Aspirin on Preventing Vascular Access Dysfunction in Incident Hemodialysis Patients: A Prospective Cohort Study in Korean Clinical Research Centers for End-Stage Renal Disease (CRC for ESRD)
Source: J Clin Med. 2019 May 14;8(5):677. doi: 10.3390/jcm8050677 (PMC6572336; doi:10.3390/jcm8050677)
Supplement: Supplementary file 1 [file jcm-08-00677-s001.pdf]

Supplementary table S1. Patients who were not using AVF or AVG within three months from the HD initiation

**Total (n = 151)**	
Anticipating kidney transplantation	67 (44.4%)
Poor general condition & short life expectancy	41 (27.2%)
Delayed AVF/AVG operation	35 (23.2%)
Extra-reasons	8 (5.3%)

Abbreviations; AVF, arteriovenous fistula; AVG, arteriovenous graft; HD, hemodialysis

Supplementary table S2. Baseline clinical characteristics and biochemical variables of the study population according to baseline aspirin use after propensity score matching

**Variables**	**Total (n = 480)**	**Aspirin users (n = 240)**	**Aspirin non-users (n = 240)**	**P-value**
Age, years	62.7±11.4	62.6±11.3	62.8±11.5	0.850
Male, n (%)	321 (66.9%)	160 (66.7%)	161 (67.1%)	1.000
Body mass index, kg/m²	23.3±3.5	23.2±3.3	23.4±3.7	0.456
Comorbid diseases, n (%)				
Diabetes mellitus	349 (72.7%)	176 (73.3%)	173 (72.1%)	0.838
Coronary arterial disease	106 (22.1%)	81 (33.8%)	25 (10.4%)	<0.001
Peripheral arterial disease	57 (11.9%)	40 (16.7%)	17 (7.1%)	0.002
Cerebrovascular accident	20 (4.2%)	12 (5.0%)	8 (3.3%)	0.494
Smoking, n (%)				
Non-smoker	237 (49.4%)	112 (46.7%)	125 (52.1%)	0.273
Ex- or Current smoker	243 (50.6%)	128 (53.3%)	115 (47.9%)	
Biochemical parameters				
Hemoglobin, g/dL	9.0±1.6	9.1±1.6	9.0±1.6	0.388
Albumin, g/dL	3.3±0.6	3.4±0.6	3.3±0.5	0.789
Blood urea nitrogen, mg/dL	84.3±35.3	82.4±35.0	86.2±35.5	0.241
Creatinine, mg/dL	8.4±3.8	8.3±3.3	8.6±4.3	0.071
Total cholesterol, mg/dL	152.5±45.3	146.5±43.1	158.6±46.7	0.006
Triglyceride, mg/dL	125.0±70.9	121.2±71.3	129.1±70.4	0.291
Arteriovenous fistula, n (%)	377 (78.5%)	182 (75.8%)	195 (81.2%)	0.182
Vascular access failure event (primary outcome)‡, n (%)	110 (22.9%)	52 (21.7%)	58 (24.2%)	0.587
Follow-up duration, months†	28 (9-49)	26 (8-47)	31 (10-51)	0.143
Incidence rate, person-year	0.091	0.090	0.091	0.968

Data are expressed as mean ± SDs or numbers (%) except where noted. †Median (interquartile range). ‡The primary outcome was defined by the first AVF/AVG intervention or salvage procedure, including percutaneous transluminal angioplasty or surgery for vascular access failure. SD = standard deviation; AVF = arteriovenous fistula; AVG = arteriovenous graft.
